# Searching for Chymase Inhibitors among Chamomile Compounds Using a Computational-Based Approach

**DOI:** 10.3390/biom9010005

**Published:** 2018-12-21

**Authors:** Amit Dubey, Serena Dotolo, Pramod W. Ramteke, Angelo Facchiano, Anna Marabotti

**Affiliations:** 1 Istituto di Scienze dell’Alimentazione—CNR, via Roma 64, 83100 Avellino, Italy; ameetbioinfo@gmail.com (A.D.); serenadotolo@hotmail.it (S.D.); 2 Indian Institute of Information Technology, Allahabad 211015, India; 3 National AIDS Research Institute, Pune 411026, India; 4 Jacob School of Biotechnology and Bioengineering, Sam Higginbottom Institute of Agriculture, Technology and Sciences, Allahabad 211007, India; pwramteke@yahoo.com; 5 Dipartimento di Chimica e Biologia “A. Zambelli”, Università degli Studi di Salerno, Via Giovanni Paolo II 132, 84084 Fisciano, Italy

**Keywords:** chymase, cardiovascular diseases, chamomile, chlorogenic acid, matricin, pharmacophore, docking, molecular dynamics simulations

## Abstract

Inhibitors of chymase have good potential to provide a novel therapeutic approach for the treatment of cardiovascular diseases. We used a computational approach based on pharmacophore modeling, docking, and molecular dynamics simulations to evaluate the potential ability of 13 natural compounds from chamomile extracts to bind chymase enzyme. The results indicated that some chamomile compounds can bind to the active site of human chymase. In particular, chlorogenic acid had a predicted binding energy comparable or even better than that of some known chymase inhibitors, interacted stably with key amino acids in the chymase active site, and appeared to be more selective for chymase than other serine proteases. Therefore, chlorogenic acid is a promising starting point for developing new chymase inhibitors.

## 1. Introduction

Human chymase enzyme (EC 3.4.21.39) is a chymotrypsin-like serine protease catalyzing the hydrolysis of peptide bonds. It is stored in a latent form in secretory granules of mast cells. Upon injury or inflammation, mast cells degranulate and release an active form of chymase into the local tissue. This enzyme binds angiotensin I with a very high affinity at the same site as angiotensin-converting enzyme (ACE), and converts angiotensin I to angiotensin II at a substantially greater rate than ACE does [1]. Moreover, a recent study identified chymase as the primary enzyme accounting for the formation of angiotensin II from the dodecapeptide angiotensin-(1-12), an extended form of angiotensin I, in the human heart [2]. Considering that cardiovascular diseases are still the leading cause of death in the U.S. and in developed countries in general, and that the control of hypertension greatly reduces the risk of stroke and heart failure [3], treatment with chymase inhibitors during the acute phase of myocardial infarction represents a potentially useful strategy in reducing cardiac dysfunction, involving targets other than ACE suppression [4]. Indeed, in animal models, the use of chymase inhibitors has shown improvement in cardiovascular remodeling, without affecting systemic cardiovascular homeostasis [5]. Therefore, there is an interest in developing specific chymase inhibitors for their use, either alone or in combination with other agents, as new therapeutic regimens for cardiovascular diseases [4]. During the last 20 years, several known synthetic peptide and nonpeptide inhibitors of chymase have been synthesized [6,7,8,9,10], and also computational approaches have been used to find new inhibitors, by applying different procedures to design or to search for suitable molecules among different databases of chemical compounds [11,12,13]. However, the search for new inhibitors is still ongoing, and new fields can be explored to find active compounds.

Herbal drugs have been used for thousands of years in Eastern countries as conventional medicines, and in the last decades, they have gradually entered also into Western medical practices [14]. The analysis of their properties using computational technologies can provide biological evidence for basic understandings of molecular mechanisms, safety, and efficacy [15]. Starting from this consideration, we decided to search for possible chymase inhibitors in databases of natural or biogenic-like compounds by applying a computational approach. Recently, some promising molecules have been found with a predicted binding energy better than that of several known chymase inhibitors, and they also showed selectivity toward chymase with respect to other serine proteases [16]. Additionally, searching for possible chymase inhibitors among the active components of Ginkgo biloba extract (the most commonly reported herbal supplement used in Western countries, especially in the elderly [17]), two other promising molecules (ginkgolic acid and proanthocyanidin) were predicted to be suitable for their possible use as chymase inhibitors [18]. Therefore, we decided to look for other possible promising molecules from other natural sources.

Another very popular herb, also used for medicinal purposes in Western countries, is chamomile, whose standardized tea and herbal extracts are prepared from dried flowers of the *Matricaria* genus. Chamomile is one of the oldest, most widely used, and well-documented medical plants in the world, and probably its use will continue in the future because it contains various bioactive phytochemicals that could provide potential health benefits [19]. In fact, the essential oil from the flowers contains several terpenoids such as alpha-bisabolol and its oxides; flavonoids and other phenolic compounds such as apigenin, quercetin, patuletin, luteolin and their glucosides; coumarins such as herniarin and umbelliferone; ferulic and caffeic acid; and chlorogenic acid [20,21,22].

The therapeutic use of chamomile for a variety of healing applications has been based essentially on popular medicine, with little scientific evidence. However, several scientific reports and experiments conducted in in vitro and in vivo models (including human studies) are available in the literature and have supported the evidence of not only its well-known mild sedative and anxiolytic effects, but also of its anti-inflammatory and antiphlogistic properties, as well as of its antimicrobial, antioxidant, and antitumoral properties (reviewed in Reference [19]). In the past, it has been shown that oral ingestion of chamomile tea produces significant hemodynamic changes in cardiovascular patients [23]. Other epidemiological studies have reported that the intake of those flavonoids particularly present in chamomile is inversely associated with heart disease risk [24,25,26]. Therefore, active compounds in chamomile can have an influence on metabolic pathways related to cardiovascular diseases, perhaps interacting with specific targets involved in heart activity.

The goal of our present study was the search for novel inhibitors of chymase enzyme among different chamomile active compounds, by structure-based pharmacophore modeling, docking, and molecular dynamics (MD) simulations. In this way, the activity of compounds derived from traditional phytotherapy was interpreted by means of innovative drug screening techniques.

## 2. Materials and Methods

### 2.1. Structure-Based Pharmacophore Models and Ligand Screening

The development and validation of structure-based pharmacophore models for chymase was made by the Receptor-Ligand Pharmacophore Generation protocol of Discovery Studio (Dassault Systèmes BIOVIA, Discovery Studio Modeling Environment, Release 4.5, San Diego, CA, USA, 2015), using as reference the crystal structure of human chymase complexed to the inhibitor 2-[3-({methyl[1-(2-naphthoyl)piperidin-4-yl]amino}carbonyl)-2-naphthyl]-1-(1- naphthyl)-2-oxoethylphosphonic acid (OHH) from the RCSB Protein Data Bank (PDB) [27] (PDB code 1T31 [7]), as described in our previous work [16]. The 3D structures of 13 active compounds from chamomile extract (alpha-bisabolol, alpha-farnesene, alpha-pinene, bisabolol, caffeic acid, chamazulene, chlorogenic acid, herniarin, matricin, nobilin, patuletin, salicylic acid, and umbelliferone) available from the PubChem database [28] were downloaded and mapped against the 10 pharmacophore models developed in our previous work [16], using Discovery Studio to evaluate their matches according to the pharmacophore features identified previously.

### 2.2. Molecular Docking Simulations

The binding mode of the 13 compounds listed above inside the active site of the chymase enzyme was investigated by docking simulations. Three different programs were applied to this system: AutoDock version 4.2, setting up the system with ADT 1.5.6 software [29]; Glide release 2015-3 (Schrodinger LLC, New York, NY, USA) [30]; and Molegro Virtual Docker (MVD) version 2013.6.0 (Qiagen Bioinformatics) [31]. The structure of chymase used to develop the structure-based pharmacophores was used also for this application. The structure of the inhibitor OHH, extracted from the PDB file, was used as an internal control to perform a self-docking test, in order to check for the correctness of the parameters used and to provide an estimation of its binding energy. Additionally, the structure of another known chymase inhibitor, methyllinderone [11], was downloaded from the PubChem database and used as a control in docking procedures to provide a comparison of the predicted binding energies with respect to those obtained for chamomile compounds. Furthermore, other proteins sharing an enzymatic activity similar to chymase, namely kallikrein (PDB code: 1LO6) [32], tryptase (PDB code: 2FPZ) [33], and elastase (PDB code: 5ABW) [34] were used to investigate the selectivity of these compounds toward chymase.

Docking simulations with AutoDock were set up as described in our previous studies [16,18]. For Glide, the structures were prepared using Protein Preparation Wizard of Maestro graphical user interface (Schrodinger LLC, New York). Hydrogens were added, ionization and tautomeric states were generated by Epik [35], and proton orientations were set by PROPKA [36]. The active site grid was generated based on the already co-crystallized ligand using a receptor grid generation module. The van der Waals radii of nonpolar receptor atoms (partial charge cut-off 0.25) were scaled down to 0.8 Å. Ligands were docked at standard precision, post-docking minimization was enabled, Epik state penalties were added to docking scores, and a maximum of 100 poses were recorded per ligand. Two different scores were assigned to the result. The GlideScore is an empirical scoring function that approximates the ligand binding free energy. It has many terms, including force field (electrostatic, van der Waals) contributions and terms rewarding or penalizing interactions known to influence ligand binding. It has been optimized for docking accuracy, database enrichment, and binding affinity prediction, and should be used to rank poses of different ligands. More negative values represent tighter binders. The Glide Emodel has a more significant weighting of the force field components (electrostatic and van der Waals energies), which makes it well-suited for comparing conformers, but much less so for comparing chemically distinct species. Therefore, Glide uses Emodel to pick the “best” pose of a ligand (pose selection), and then ranks these best poses against one another with GlideScore. For MVD, the protein structures were prepared using the protein preparation wizard suitable for this program, by adding explicit hydrogens. This program automatically identifies potential binding sites (also referred to as cavities or active sites) by using its cavity detection algorithm. The potential binding site was predicted, and docking was performed within cavity 1, having a volume of 35.84 A^3^ and a surface area of 148.48 A^2^ centered on the experimentally known ligand position. The chamomile compounds were imported into MVD the workspace. The bonds flexibility of the ligands was set, and the side chain flexibility of the amino acids in the binding cavity was set with a tolerance of 1.10 and a strength of 0.90 for docking simulations. The root mean square deviation (RMSD) threshold for multiple cluster poses was set at <2.00 Å. MolDock SE was selected as a search algorithm. The population size was set at 50 with an energy threshold of 100. The docking algorithm was set at a maximum iteration of 1500 with a simplex evolution size of 300 and a minimum of 100 runs. The meaning of the MolDock score and of the Rerank score are analogous to the Glide Score and Glide Emodel, respectively.

The conformations representative of the best poses obtained for each compound tested and each program were selected, saved in .pdb format, and analyzed for their interactions with the enzyme by using the tools available in Discovery Studio. A superposition was also performed between the co-crystallized ligand of chymase and chamomile compounds to show the reciprocal position in the active site.

### 2.3. Molecular Dynamics Simulations

50-ns-long MD simulations were performed with the parallel MD program Desmond (Schrodinger LLC, New York) running on a high-performance Linux cluster computer. The four complexes between chymase and OHH, chlorogenic acid, matricin, and methyllinderone (obtained from the docking simulations performed with AutoDock 4.2) were optimized with the protein preparation wizard in Maestro by assigning bond orders, adding hydrogen, and correcting wrong bond types. In Desmond, the volume of space in which the simulation took place (the global cell) was divided into regular 3D simulation boxes of 10 Å × 10 Å × 10 Å. Each box was assigned to a single Desmond process. These boxes constituted the total simulation space, with a total volume of 90 Å × 90 Å × 90 Å. A predefined TIP3P water model was used to simulate water molecules. Orthorhombic periodic boundary conditions were set up to specify the shape and size of the repeating unit buffered at 10 Å distances. In order to neutralize the system, appropriate counterions Na^+^/Cl^−^ were added to balance the system charge and were placed randomly in the solvated system. Following this, minimization and relaxation of each protein–ligand complex was performed using the default 8-stages protocol of Desmond, including NVT and NPT short equilibration stages. After the equilibration steps, MD simulations were carried out with the periodic boundary conditions in the NPT ensemble using OPLS 2005 force field parameters [37]. The pressure was kept constant at 1 bar, and the temperature was kept at 300 K, using a Nosé–Hoover chain [38] and Martyna–Tobias–Klein methods [39]. The short-range and long-range Coulombic interactions were calculated with a cut-off radius of 9 Å and with the smooth particle mesh method [40] (Ewald tolerance: 1.0 × 10^−9^). The M-SHAKE algorithm [41] was used to constrain bonds containing hydrogen atoms. All the simulations used a multistep RESPA integrator [42] with a 2.0-fs time step for bonded interactions and short-range nonbonded interactions and a 6.0-fs time step for long-range nonbonded interactions. During MD, the energies were recorded every 1.2 ps, while the coordinates were recorded every 9.6 ps. The simulations were analyzed using the Desmond program of the Schrödinger suite of programs. The RMSD from the initial structure and the root mean square fluctuations (RMSFs) were calculated for all MD simulations. All atoms were included in the calculations of the RMSF. Atomic distances important for ligand binding and structural changes in chymase were calculated for all the simulations.

## 3. Results

### 3.1. Structure-Based Pharmacophore Modeling and Ligand Screening

**Figure 1:**
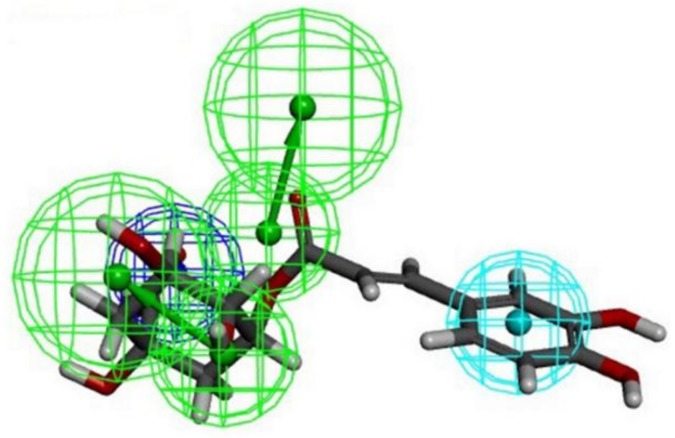
Fitting of chlorogenic acid onto pharmacophore 10. The colored spheres identify the position and the type of the features: Green sphere = hydrogen bond acceptor; cyan = hydrophobic; blue = negative ionizable.

Pharmacophore-based screening of putative chymase ligands was performed onto the subset of 13 compounds from chamomile extract against the 10 pharmacophore models obtained, as described previously [16]. Chlorogenic acid was the only compound that mapped the features of these pharmacophores. In particular, this compound fit the features of pharmacophore 10 with an acceptable fit score (0.723, calculated with the Ligand Profiler protocol), and the features of pharmacophores 7 and 9 with very low fit values (<0.1). Pharmacophore 10 was indicated as the one with the best sensitivity but the lowest specificity in our previous work [16]. Figure 1 shows the correspondence of chlorogenic acid with the set of features identified by pharmacophore 10. No correspondence was found, however, with the features identified by pharmacophore 2 used in our previous study to identify possible chymase ligands among natural biogenic compounds [16]. Probably for this reason, this compound was not identified as a suitable chymase inhibitor in our previous study.

### 3.2. Molecular Docking Simulations

All 13 selected compounds from chamomile extract were used to perform molecular docking simulations toward the structure of human chymase. In order to improve the reliability of the predictions, three different tools were used, and their results were compared (Table 1). All of these compounds showed predicted negative binding energies, indicating potentially favorable interaction with the enzyme. These predicted binding energies were in all cases higher than that of the reference compound OHH, suggesting a lower affinity of these compounds for the enzyme. The three programs agreed in predicting chlorogenic acid among the best ligands for chymase. In particular, the binding energy predicted by Glide for chlorogenic acid was very similar to the one predicted for the crystallographic inhibitor OHH.

Unexpectedly, all three programs agreed in predicting high (unfavorable) binding energies for another known chymase inhibitor, methyllinderone (Table 1), whereas AutoDock and MVD agreed in predicting the highest affinity toward chymase for matricin (in both cases, chlorogenic acid was ranked second). The three programs agreed also in identifying alpha-pinene among the worst interactors for chymase. The ranking of the other compounds was rather variable across the three different docking simulations, but it is possible to note that there was in general more agreement between AutoDock and MVT results than with Glide.

The full analysis of the interactions in the complexes between chymase and active compounds from chamomile is reported in Table S1. In particular, the complexes between chymase and matricin or chlorogenic acid, obtained by docking with the three different programs, showed that both of these compounds were predicted to interact with several key residues constituting the active site of the enzyme (Lys40, His57, Asp102, Phe191, Lys192, Gly193, Ser195, Tyr215, Gly216, Arg217, and Ala226).

**Figure 2:**
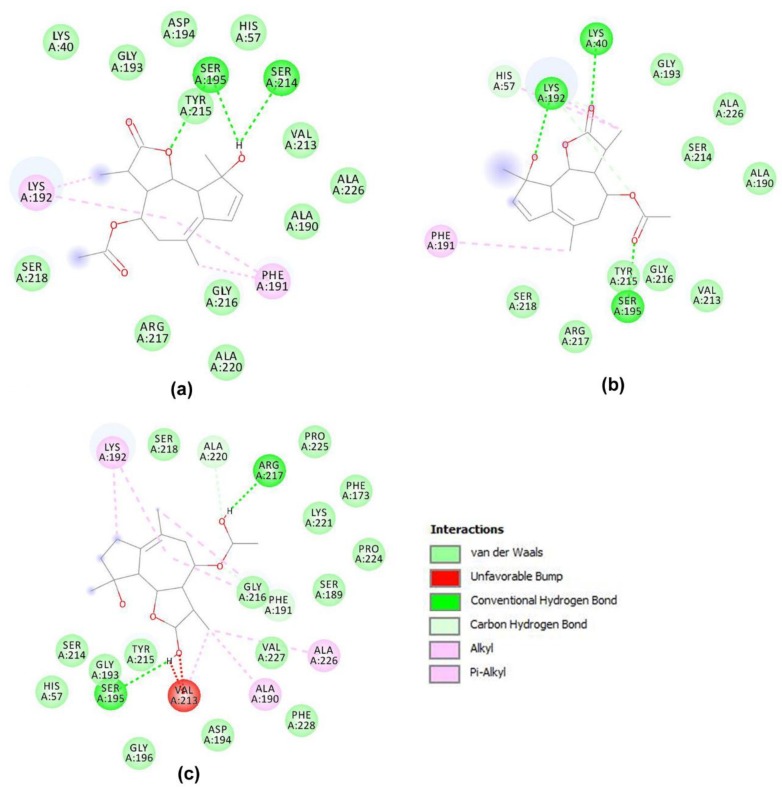
Scheme of the interactions between matricin and selected residues of the chymase active site. The complexes were obtained using: (**a**) AutoDock, (**b**) Glide, and (**c**) Molegro Virtual Docker (MVD). The legend in the picture describes the different kinds of interactions of the molecule with the residues of the enzyme, identified by Discovery Studio.

The analysis of the representative pose for matricin obtained with AutoDock showed that this compound established strong hydrogen bonds with Ser195, and additionally with Ser214, and interacted with a ring aromatic group establishing hydrophobic interactions with Phe191 and Lys192 (Figure 2a and Table S1). In the complex obtained with Glide, additionally Lys40 and His57 were able to interact with this compound with a hydrogen bond each (Figure 2b and Table S1). Finally, in the complex obtained with MVD, the interaction of matricin with chymase involved again Phe191, Lys192, and Ser 195, and additionally also Arg217 (with a strong hydrogen bond) and Ala226 (with a weak bond). Other interactions were also made with Ala190, Val213, and Ala220 (Figure 2c and Table S1).

**Figure 3:**
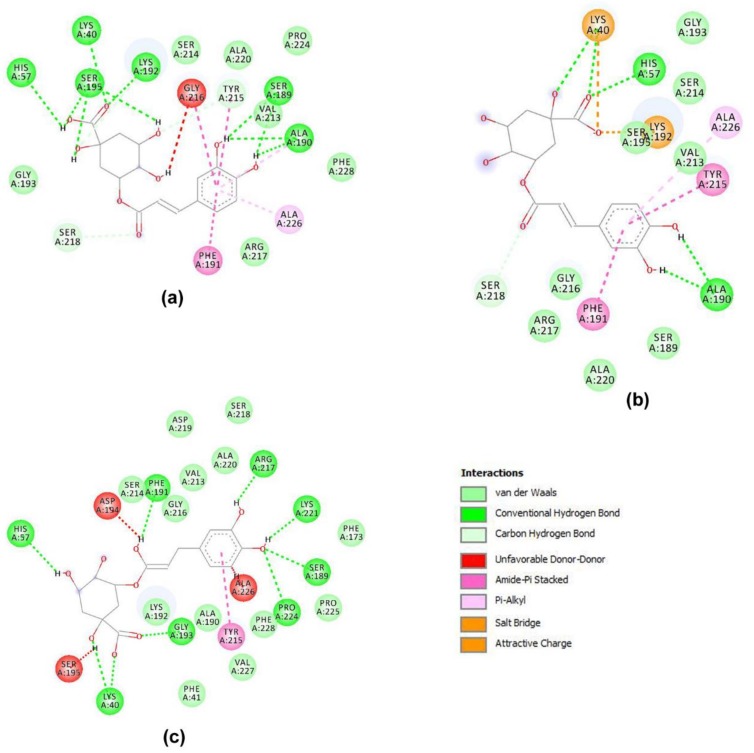
Scheme of the interactions between chlorogenic acid and selected residues of the chymase active site. The complexes were obtained using: (**a**) AutoDock, (**b**) Glide, and (**c**) MVD. The legend in the picture describes the different kinds of interactions of the molecule with the residues of the enzyme, identified by Discovery Studio.

The interaction of chlorogenic acid with the chymase appeared to involve many more residues of the active site in each of the three complexes obtained with AutoDock, Glide, and MVD. In all cases, it was possible to predict that this compound makes several hydrogen bonds with Lys40, His57, and Lys 192. Other interactions in common among the three simulations were those with Tyr215 and Phe191 via their ring aromatic groups establishing hydrophobic interactions. Chlorogenic acid also showed some unfavorable interactions with residues involved in hydrogen bonds (Figure 3 and Table S1).

In order to understand why matricin was not identified as a potential ligand for chymase by the preliminary pharmacophore analysis, a further ligand features mapping analysis was performed on the complexes between matricin/chlorogenic acid/OHH and chymase. From this analysis, it was evident that the matricin shared only 21 pharmacophore features belonging to three different categories (HB_DONOR, HB_ACCEPTOR, HYDROPHOBIC) with the pharmacophore 10 developed from the complex chymase/OHH, whereas chlorogenic acid shared 38 pharmacophore features belonging to five different categories (HB_DONOR, HB_ACCEPTOR, HYDROPHOBIC, NEG_IONIZABLE, and RING_AROMATIC) (Table S2). Therefore, the total number of features in matricin was considerably lower with respect to chlorogenic acid, and this could be the reason why matricin was not selected by the pharmacophore screening procedure. However, it is possible to note that the features essential for a good interaction with the enzyme were conserved in both cases, and this could justify the fact that matricin was predicted to bind chymase with a good binding energy.

**Figure 4:**
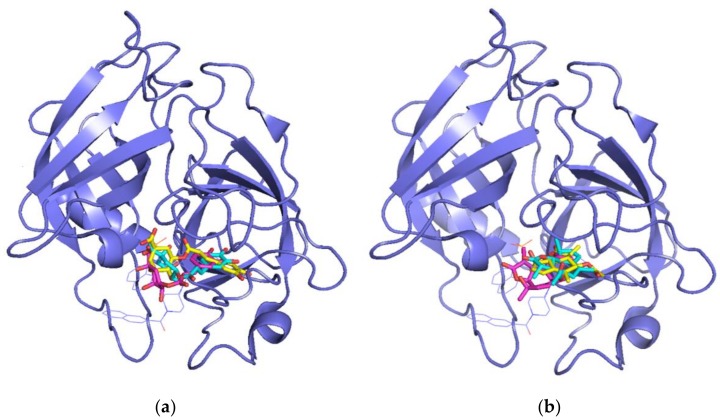
Superposition of the structure of chymase co-crystallized ligand OHH (thin line, blue color) with the best poses for: (**a**) Chlorogenic acid and (**b**) matricin in the complexes obtained with AutoDock (cyan), Glide (magenta), and MVD (yellow). The ligand docked into the active site is represented in stick mode.

We superimposed the poses obtained for the matricin and chlorogenic acid compounds by the three docking approaches onto the crystallographic inhibitor OHH in the chymase to compare their relative positions. In all cases, both chlorogenic acid (Figure 4a) and matricin (Figure 4b) occupied the right side of the catalytic pocket, approximately superimposed onto the central naphthalene ring of OHH. Interestingly, the quinic acid moiety of chlorogenic acid was superimposed onto the phosphate moiety of the OHH inhibitor, indicating that an acidic group seemed to be necessary to interact with the enzyme in that position. This was in line with the previously discussed evidence that the negative ionizable feature found in pharmacophore 10 seemed to be the most important one for the maximum fit score with chlorogenic acid.

In order to evaluate the selectivity of these two compounds toward human chymase, we also performed a docking toward three other enzymes belonging to the hydrolase family (i.e., kallikrein, tryptase, and elastase). The results are shown in Table 2. Comparing the data to Table 1, it is possible to note that in the simulations performed with AutoDock and MVD, both chlorogenic acid and matricin showed a predicted affinity higher for chymase than for the other three enzymes. Moreover, chlorogenic acid and matricin were consistently predicted by the three tools to be more selective toward tryptase and elastase than toward kallikrein. On the contrary, the simulations with Glide did not highlight significant differences between the affinities of these compounds for these different enzymes. However, even Glide predicted that chlorogenic acid tended to bind less preferably to tryptase and elastase, whereas matricin was predicted to have a higher affinity for these three enzymes than for chymase itself. The predictions made by Glide on the co-crystallized inhibitor OHH showed no difference in affinity for the other hydrolases, whereas AutoDock and MVT predicted a difference in binding energy between chymase and the other three enzymes. Overall, it is hard to state if these differences were significant or not, but the fact that two predictors agreed in finding differences of the same trend could support the hypothesis that these molecules had at least a partial selectivity for human chymase.

### 3.3. Molecular Dynamics Simulations

The crystallographic structure of OHH bound to chymase and the complexes of the same protein with chlorogenic acid, matricin, and methyllinderone obtained by docking were submitted to 50 ns of MD simulation, in order to assess the stability of the ligand into the binding pocket of the protein.

**Figure 5:**
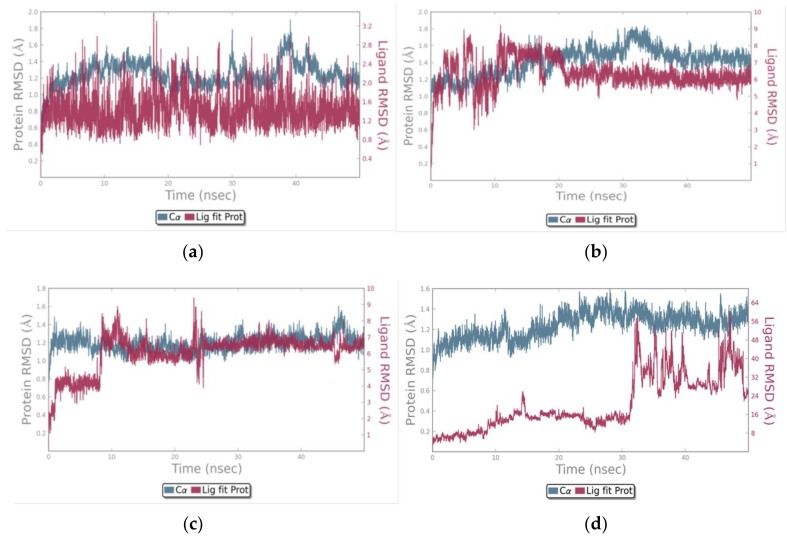
Evolution of the Root mean square deviation (RMSD) during MD simulations of chymase in complex with the different ligands: (**a**) Crystallographic ligand OHH; (**b**) chlorogenic acid; (**c**) matricin; (**d**) methyllinderone. The first frame was used as the reference, and it was regarded as time *t* = 0. On the left axis, the protein RMSD value (represented as the blue trace in the graph) is shown, whereas on the right axis, the ligand RMSD value (represented as the red trace in the graph) is shown. Values are reported in Å. The graphs were produced by Desmond analysis tools.

The calculation of the RMSD of the Cα atoms of the chymase in the four different complexes (Figure 5, blue traces) showed that the protein structure was quite stable in all of the simulations, soon reaching equilibrium around an RMSD value of approximately 1.2–1.3 Å with respect to the starting conformation. No significant variation was detected either in the percentage of secondary structure elements or in the RMSF of the protein backbone among the four complexes (data not shown). On the contrary, the RMSD calculated on the different ligands with respect to the protein showed significant differences (Figure 5, red traces). In the crystallographic complex, the position of OHH in the active site of chymase was practically fixed for all of the simulation, and the RMSD oscillated around an average value of about 1.5 Å. This was expected, considering the high affinity of this compound for the chymase [7]. For chlorogenic acid and matricin, the fluctuation was higher, and the RMSD reached a final value of about 6 Å in both cases.

**Figure 6:**
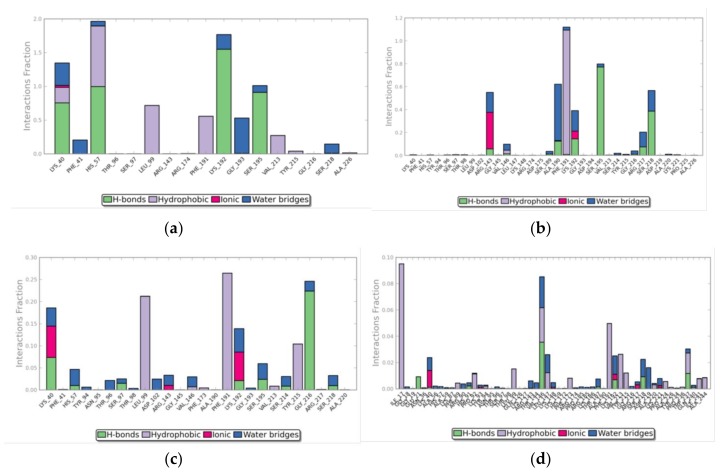
Evolution of protein–ligand contacts during MD simulations. The bar chart shows the percentage and the type of the interactions between chymase and (**a**) crystallographic ligand OHH, (**b**) chlorogenic acid, (**c**) matricin, and (**d**) methyllinderone. On the *x* axis, the residues involved in the interactions are reported, and on the *y* axis, the normalized value of the temporal length of the interactions during the simulation is reported. A value higher than 1 indicates that the protein residue could make multiple contacts of the same subtype with the ligand. The color code associated to the type of the interaction is shown under the graph. The graphs were produced by the Desmond analysis tool.

However, looking at the ligand RMSF (Figure S1), it is possible to note that, for chlorogenic acid, the distance between a portion of the molecule (atoms 12 to 24) and the protein was kept on average at around 1 Å (Figure S1B). Thus, this compound was able to move into the active site, changing its position compared to the starting one, without losing contact with the protein. The RMSF calculated for matricin (Figure S1C) was somewhat higher, but still compatible with interactions kept in the protein’s active site. It is worth noting that OHH was a relatively big ligand, with 49 heavy atoms (almost double compared to both chlorogenic acid and matricin), it fully occupied the active cavity in the pocket, and therefore its mobility was forcedly limited (Figure S1A). On the contrary, these two ligands, being smaller, could move into the active site with more freedom, and this was probably the reason why the predicted affinity of these two ligands for chymase was lower than that of the co-crystallographic inhibitor. Unexpectedly, the known inhibitor methyllinderone showed a very large RMSD and, after about 30 ns of simulation, this value increased up to 30–40 Å, a value compatible with the exit from the active site cavity. In addition, the average RMSF for this compound was higher than 20 Å (Figure S1D). Frames of the trajectory taken every 5 ns confirmed this phenomenon. OHH was fixed in the active site, and chlorogenic acid and matricin rotated and moved in the cavity without exiting out of it. The molecule of methyllinderone loosened the contacts with the active site residues after about 20 ns of simulation, and was then disperded in the solvent, far from the protein (Figure S2). The comparison of the contacts of the ligands during the simulations showed that OHH interacted for a large fraction of the simulation with those residues already identified as part of the active site, in particular with Lys40, His57, Lys192, and Ser195 for a fraction higher than 1 (Figure 6a). The interactions involved were essentially hydrogen bonds and, to a lower extent, hydrophobic contacts, but also water bridges contributed to the binding. Due to their higher motility, the contacts of chlorogenic acid (Figure 6b) were distributed over a larger number of residues, but it is possible to note that interactions with Ala190, Phe191, and Ser195 were present for more than 50% of the simulation time, with also a contribution of about 40% of contacts with Lys192. On the contrary, the contacts with Lys40 and His57 were irrelevant during the simulation. Similarly to OHH, the interaction with Phe191 was predominantly of a hydrophobic nature, whereas that with Ser195 was made mainly by hydrogen bonds. Instead, the interaction with Lys192 (which was mainly formed by hydrogen bonds in OHH), resulted from a composition of hydrogen bonds, ionic interactions, and water bridges. For matricin (Figure 6c), the fraction of interactions was always lower than 30% of simulation time: However, it is possible to note that contacts with active site residues Lys40, Phe191, and Gly216 were present for about 20% of the simulation. Finally, the exit of methyllinderone from the active site was in line with the observation that none of its interactions accounted for more than 10% of simulation time (Figure 6d).

Moreover, the contacts were extremely distributed along the protein sequence, indicating a lack of their specificity during the simulation time. The timeline of the contacts (Figure S3) showed a clear persistence of contacts for OHH and, to a lesser extent, for chlorogenic acid, whereas the contacts of matricin were less defined and specific, but still present, and those of methyllinderone were randomly distributed.

The data obtained by the MD simulations supported the hypothesis that chlorogenic acid may act as a ligand for chymase enzyme, whereas matricin is a worse candidate for this role. Surprisingly, methyllinderone, a known chymase inhibitor, was not persistently included in the active site in our simulations.

## 4. Discussion

The present study can be of interest not only in identifying new pharmacologically active natural compounds, but also in suggesting molecular mechanisms by which these compounds can achieve their putative biological effects. Indeed, the compounds identified as potential chymase inhibitors in this work have been previously characterized for some interesting biological activities. Chlorogenic acid is a natural chemical compound belonging to the family of esters of hydroxycinnamic acids, occurring in many plants other than chamomile, such as coffee, tea, apple, black chokeberry, eggplant, peach, and prunes. Interestingly, some reviews have reported modest blood pressure lowering effects after chlorogenic acid administration [43,44], mainly attributed to the ability of this compound to attenuate oxidative stress, thus improving endothelial function and nitric oxide bioavailability in the arterial vasculature [43]. The interaction with chymase might be another process involved in this pharmacological activity. However, further studies will be required to definitely prove the activity of this compound as a new chymase inhibitor.

Matricin is a sesquiterpene lactone, precursor of the chamazulene, belonging to that fraction of chamomile extract considered responsible for its antiphlogistic activity [45]. To date, matricin has been considered mainly as a prodrug whose activity is related to its transformation into chamazulene, but not as having anti-inflammatory activity per se. From our results, instead, it seems that matricin could be able to interact with chymase, although this interaction seems to be less favorable than that of chlorogenic acid.

Furthermore, the most unexpected and surprising results we obtained are in reference to methyllinderone, a compound reported as a known chymase inhibitor in the literature. Indeed, this compound was reported to inhibit the human chymase in a paper by Aoayama and colleagues [11]. In that very short paper (and references therein), the inhibition constant of methyllinderone determined for human chymase was relatively high (30 μM) (as a reference, the binding affinity of OHH is reported to be 2.3 nM [7]). Moreover, those authors discussed a putative inhibition mechanism on the basis of some theoretical analyses, without effectively proving it. In the present work, this compound was not included among the best predicted ligands by any of the three docking procedures applied (rather, it was classified as the third worst one by AutoDock and as the worst one by Glide, even with a positive score). Additionally, MD simulations predicted the exit of this compound from the active site after about 20 ns of simulation, supporting the idea that this compound is not able to form a stable complex with this enzyme. Another observation that questions the classification of this compound as an inhibitor of chymase enzyme is the fact that, looking in the literature available to date, we did not find other evidence of the inhibitory activity of this compound (the search in PubMed for articles using the keywords “chymase” and “methyllinderone” gave as a single result the article by Aoayama and colleagues [11]). Thus, we think that more evidence must be reported on the activity of methyllinderone to support its alleged activity as a chymase inhibitor.

## 5. Conclusions

The present work aimed to discover novel inhibitors of chymase enzyme from natural sources, which selectively bind to its active site. Using previously developed pharmacophore models based on the X-ray structure of the enzyme bound to a well-known inhibitor, chlorogenic acid was identified among the set of chamomile compounds as the one with a potential ability to bind to chymase. The following docking approach was able to identify matricin as another compound potentially able to bind this enzyme. Additionally, MD simulations supported the hypothesis that chlorogenic acid may bind stably to the protein, keeping contacts with the residues in the active site, whereas the result for matricin was not completely convincing. However, the fact that matricin was able to persist in the cavity of chymase and to contact some residues of the active site suggests that modifications to this compound in order to improve its specificity and affinity might allow identification of a new family of chymase inhibitors. We hope therefore that our present study will be a first step toward the development of novel chymase inhibitors for the treatment of cardiovascular diseases.

## Figures and Tables

**Table 1:** Results of molecular docking simulations of chamomile compounds into the active site of chymase. The best-predicted binding energy corresponded to that of the representative pose of the cluster of solutions found. The value of the predicted binding energy of the representative pose and of the number of poses of the corresponding cluster for the crystallographic inhibitor OHH (obtained previously) [16]) and for methyllinderone (this study) are reported (in italics) for comparison.

	AutoDock		Glide		Molegro Virtual Docker	
Chamomile Compounds (PubChem ID)	Best-Predicted Binding Energy (kcal/mol)	No. of Poses in the Cluster with Best-Predicted Energy	Glide Score	Glide Emodel	Moldock Score	Rerank Score
Alpha-Bisabolol (10586)	−8.28	85	−4.915	−32.667	−109.629	−92.408
Alpha-Farnesene (5281516)	−7.57	72	−1.071	−26.181	−120.466	−97.4505
Alpha-Pinene (6654)	−5.35	100	−4.194	−18.417	−49.1045	−45.0675
Bisabolol (1549992)	−8.27	85	−4.800	−33.463	−110.496	−93.6185
Caffeic Acid (689043)	−6.62	76	−6.192	−60.275	−115.909	−98.1313
Chamazulene (10719)	−8.43	100	−5.294	−32.017	−124.609	−75.8608
Chlorogenic Acid (1794427)	−8.57	28	−7.037	−79.332	−138.276	−10.5578
Herniarin (10748)	−6.9	100	−5.809	−37.800	−96.5088	−77.6553
Matricin (92265)	−9.12	85	−5.040	−37.582	−139.206	−48.1241
Nobilin (11953937)	−7.9	95	−4.835	−41.410	−129.981	−37.3249
Patuletin (5281678)	−7.44	36	−5.969	−53.612	−120.525	−53.6831
Salicylic Acid (338)	−5.26	76	−5.832	−44.952	−82.5318	−66.5609
Umbelliferone (5281426)	−6.72	82	−5.868	−37.074	−90.6394	−70.215
*OHH (self-docking with crystallographic inhibitor)*	*−14.84*	*90*	*−7.101*	*−78.669*	*−209.86*	*−105.051*
*Methyllinderone (21953547)*	*−6.62*	*43*	*0.469*	*−22.132*	*−114.237*	*−93.903*

**Table 2:** Analysis of the predicted specificity of selected chamomile compounds chlorogenic acid and matricin toward other hydrolases. The value of the predicted binding energy of the representative pose, and the number of poses of the corresponding cluster, for the crystallographic inhibitor OHH, obtained previously [16], are reported (in italics) for comparison.

Protease (with PDB ID) and Best Chamomile Compounds (with PubChem ID)	AutoDock		Glide		Molegro Virtual Docker	
	Best-Predicted Binding Energy (kcal/mol)	No. of Poses in the Cluster with Best-Predicted Energy	Glide Score	Glide Emodel	Moldock Score	Rerank Score
Kallikrein (1LO6)						
Chlorogenic Acid (1794427)	−7.94	32	−7.111	−69.452	−116.975	−108.579
Matricin (92265)	−8.51	75	−6.618	−46.227	−126.84	−21.8832
*Chymase Crystallographic Inhibitor OHH*	*−10.03*	*21*	*−7.439*	*−68.327*	*−174.073*	*−3.06207*
Tryptase (2FPZ)						
Chlorogenic Acid (1794427)	−6.24	41	−6.985	−59.835	−105.543	−84.9801
Matricin (92265)	−6.74	76	−5.863	−37.119	−117.111	−109.58
*Chymase Crystallographic Inhibitor OHH*	*−9.77*	*29*	*−7.441*	*−76.763*	*−157.822*	*6.93001*
Elastase (5ABW)						
Chlorogenic Acid (1794427)	−6.25	20	−6.301	−54.401	−108.314	−95.7808
Matricin (92265)	−6.34	7	−5.476	−37.090	−110.134	90.7393
*Chymase Crystallographic Inhibitor OHH*	*−12.2*	*12*	*−6.237*	*−52.337*	*−153.169*	*−85.1654*

## Supplementary Materials

The following are available online at http://www.mdpi.com/2218-273X/9/1/5/s1. The file Chamomile-paper_Biomolecules_SupplMat.docx contains the following supplementary materials, Table S1: Interactions between the selected representative poses of each chamomile compound obtained for each docking program into the active site of human chymase; Table S2: List of the feature mapping calculations performed on matricin, chlorogenic acid, and OHH (for comparison) in complex with chymase; Figure S1: Ligand RMSF, measured on the ligand heavy atoms, of (A) OHH, (B) chlorogenic acid, (C) matricin, and (D) methyllinderone, compared to the starting structure; Figure S2: Snapshots of the positions of the ligands during the simulation for (A) OHH, (B) chlorogenic acid, (C) matricin, (D) methyllinderone; Figure S3: Timeline of protein–ligand contacts during the simulation for (A) OHH, (B) chlorogenic acid, (C) matricin, (D) methyllinderone.
