# Supplementary material for: Searching for Chymase Inhibitors among Chamomile Compounds Using a Computational-Based Approach
Source: Biomolecules. 2018 Dec 21;9(1):5. doi: 10.3390/biom9010005 (PMC6358779; doi:10.3390/biom9010005)
Supplement: Supplementary file 1 [file biomolecules-09-00005-s001.pdf]

Supplementary Materials: Searching for Chymase Inhibitors among Chamomile Compounds Using a Computational-Based Approach

Amit Dubey, Serena Dotolo, Pramod W. Ramteke, Angelo Facchiano and Anna Marabotti

Table S1. Interactions between the selected representative poses of each chamomile compound obtained for each docking program into the active site of human chymase. The type of interaction is reported according to Discovery Studio classification. Residues in bold belong to the active site of the enzyme.

**Chamomile Compounds (PubChem ID)**	**AutoDock Results**	**Glide Results**	**Molegro Virtual Docker**
Alpha-Bisabolol (10586)	Cys42 (alkyl)<br>**His57 (Pi-alkyl)**<br>**Phe191 (unfavorable acceptor-acceptor, Pi-alkyl)**<br>**Ser195 (conventional H-bond)**<br>Ala226 (pi-alkyl, alkyl)	**His57 (two Pi-alkyl)**<br>**Gly216 (conventional H-bond) Ala226 (alkyl)**<br>Val213 (two alkyl)	**Lys40 (alkyl)**<br>Cys42 (alkyl)<br>**His57 (pi-alkyl)**<br>**Phe191 (two pi-alkyl)**<br>**Lys192 (alkyl)**<br>**Ser195 (unfavorable acceptor-acceptor)**<br>Ser214 (conventional H-bond)<br>**Ala226 (two alkyl)**
Alpha-Farnesene (5281516)	**Phe191 (pi-alkyl)**<br>**Lys192 (alkyl)**<br>Val213 (alkyl)<br>**Arg217 (alkyl)**<br>Ala220 (alkyl)<br>**Ala226 (alkyl)**	**His57 (two pi-alkyl)**<br>Leu99 (two alkyl)<br>Ala190 (alkyl)<br>**Phe191 (pi-alkyl)**<br>**Lys192 (alkyl)**<br>Val213 (alkyl)<br>Tyr215 (pi-alkyl)<br>**Ala226 (two alkyl)**	**Lys40 (alkyl)**<br>Cys42 (alkyl)<br>**Phe191 (pi-alkyl)**<br>**Lys192 (alkyl)**<br>**Ala226 (alkyl)**
Alpha-Pinene (6654)	**His57 (pi-alkyl)**<br>**Phe191 (pi-alkyl)**<br>**Lys192 (alkyl)**<br>**Ala226 (alkyl)**	**His57 (pi-alkyl)**<br>**Phe191(pi-alkyl)**<br>**Lys192 (two alkyl)**	**His57 (pi-alkyl)**<br>**Phe191 (pi-alkyl)**<br>**Lys192 (two alkyl)**<br>**Ala226 (alkyl)**
Bisabolol (1549992)	Cys42 (alkyl)<br>**His57 (pi-alkyl)**<br>**Phe191 (pi-alkyl)**<br>**Ser195 (conventional H-bond)**<br>**Arg217 (alkyl)**<br>**Ala226 (alkyl)**	Val146 (alkyl)<br>Ala190 (alkyl)<br>**Lys192 (alkyl)**<br>Val213 (alkyl),<br>**Ala226 (alkyl)**	**Lys40 (alkyl)**<br>Cys42 (alkyl)<br>**His57 (pi-alkyl)**<br>**Phe191 (two pi-alkyl)**<br>**Lys192 (alkyl)**<br>**Ser195 (unfavorable acceptor-acceptor)**<br>**Ser214 (conventional H-bond)**<br>**Ala226 (alkyl)**

Caffeic_Acid (689043)	**His57 (conventional H-bond)**<br>Ala190 (conventional H-bond)<br>**Phe191 (amide-pi stacked)**<br>**Lys192 (conventional H-bond)**<br>**Ser195 (conventional H-bond)**<br>**Tyr215 (amide-pi stacked)**<br>**Gly216 (amide-pi stacked)**<br>**Arg217 (conventional H-bond)**<br>**Ala226 (pi-alkyl)**	**Lys40 (salt bridge, attractive charge)**<br>**His57 (attractive charge),**<br>**Phe191 (pi-pi T-Shaped)**<br>**Lys192 (pi-alkyl, salt bridge, two attractive charges)**<br>**Gly216 (carbon hydrogen bond)**<br>**Arg217 (conventional H-bond)**	Ser189 (conventional H-bond)<br>**Phe191 (pi-pi T-shaped)**<br>**Lys192 (van der Waals)**<br>**Ser195 (conventional H-bond)**<br>**Gly216 (carbon hydrogen bond)**<br>**Arg217 (conventional H-bond)**<br>Lys221 (conventional H-bond)<br>Pro225 (conventional H-bond)
Chamazulene (10719)	**Phe191 (amide-Pi stacked, pi-alkyl)**<br>**Lys192 (pi-alkyl)**<br>Val213 (alkyl)<br>Tyr215 (amide-pi stacked)<br>**Gly216 (amide-pi stacked)**<br>**Arg217 (van der Waals)**<br>**Ala226 (alkyl)**	**His57 (pi-alkyl)**<br>**Phe191 (amide-pi stacked, pi-alkyl)**<br>**Lys192 (two pi-alkyl, alkyl)**<br>Val213 (alkyl)<br>**Tyr215 (amide-pi stacked)**<br>**Gly216 (van der Waals)**	Ala190 (two alkyl),<br>**Phe191 (two amide-pi stacked)**<br>**Lys192 (alkyl)**<br>Val213 (unfavorable bump, pi-alkyl, alkyl),<br>Ser214 (amide-pi stacked)<br>Tyr215 (amide-pi stacked),<br>**Gly216 (amide-pi stacked)**<br>**Arg217 (alkyl)**<br>**Ala226 (two pi-alkyl, two alkyl)**<br>Phe228 (pi-alkyl)

**Chlorogenic Acid (1794427)**	**Lys40 (conventional H-bond)**<br>**His57 (conventional H-bond)**<br>Ser189 (conventional H-bond)<br>Ala190 (conventional H-bond, pi-alkyl)<br>**Phe191 (amide-pi stacked)**<br>**Lys192 (conventional H-bond)**<br>**Ser195 (conventional H-bond)**<br>**Tyr215 (carbon-hydrogen bond, amide-pi stacked)**<br>**Gly216 (unfavorable donor-donor, amide-pi stacked)**<br>**Arg217 (van der Waals)**<br>Ser218 (carbon-hydrogen bond)<br>**Ala226 (pi-alkyl)**	**Lys40 (two conventional H-bond, attractive charge)**<br>**His57 (conventional H-bond)**<br>Ala190 (two conventional H-bond)<br>**Phe191 (amide-pi stacking)**<br>**Lys192 (salt bridge, attractive charge)**<br>**Tyr215 (amide-pi stacked)**<br>Gly216 (van der Waals)<br>Ser218 (carbon-hydrogen bond)<br>**Ala226 (pi-alkyl)**	**Lys40 (two conventional H-bond)**<br>**His57 (conventional H-bond)**<br>Ser189 (conventional H-bond)<br>**Phe191 (conventional H-bond)**<br>**Lys192 (conventional H-bond)**<br>**Gly193 (conventional H-bond)**<br>Asp194 (unfavorable donor-donor)<br>**Ser195 (unfavorable donor-donor)**<br>**Tyr215 (amide-pi stacked)**<br>**Gly216 (van der Waals)**<br>**Arg217 (conventional H-bond)**<br>Ser218 (carbon-hydrogen bond)<br>Lys221 (conventional H-bond)<br>Pro224 (conventional H-bond)<br>**Ala226 (unfavorable Bump)**
Herniarin (10748)	**Phe191 (amide-pi stacked)**<br>**Lys192 (van der Waals)**<br>Ser214 (amide-pi stacked)<br>**Tyr215 (amide-pi stacked)**<br>**Gly216 (van der Waals)**<br>**Arg217 (unfavorable acceptor-acceptor)**<br>Lys221 (carbon-hydrogen bond)<br>**Ala226 (pi-alkyl)**	Ala190 (carbon hydrogen bond),<br>**Phe191 (two amide-pi stacked)**<br>**Lys192 (two pi-alkyl)**<br>**Tyr215 (amide-pi stacked)**<br>**Gly216 (carbon hydrogen bond)**	Ala190(pi-alkyl)<br>**Phe191 (amide-pi stacked),**<br>**Lys192 (carbon-hydrogen bond)**<br>**Tyr215 (two amide-pi stacked)**<br>**Gly216 (van der Waals),**<br>Ala220 (carbon-hydrogen bond),<br>Lys221 (conventional H-bond)<br>**Ala226 (unfavorable Bump, pi-alkyl)**
**Matricin (92265)**	**Phe191 (pi-alkyl)**<br>**Lys192 (alkyl)**<br>**Ser195 (conventional H-bond)**<br>Ser214 (conventional H-bond)	**Lys40 (conventional H-bond),**<br>**His57 (carbon-hydrogen bond, pi-alkyl)**<br>**Phe191 (pi-alkyl)**<br>**Lys192 (conventional H-bond, carbon hydrogen bond, two alkyl)**<br>**Ser195 (conventional H-bond)**	Ala190 (alkyl)<br>**Phe191 (carbon hydrogen bond, two pi-alkyl)**<br>**Lys192 (two alkyl)**<br>**Ser195 (conventional H-bond)**<br>Val213 (alkyl, two unfavorable bump)<br>**Arg217 (conventional H-bond)**<br>Ala220 (carbon hydrogen bond)<br>**Ala226 (alkyl)**

Nobilin (11953937)	**His57 (pi-alkyl)**<br>**Phe191 (pi-alkyl)**<br>**Lys192 (conventional H-bond, alkyl)**<br>Val213 (alkyl)<br>**Gly216 (conventional H-bond)**<br>**Ala226 (alkyl)**	**His57 (pi-alkyl),**<br>Ala190 (alkyl),<br>**Phe191 (pi-alkyl),**<br>**Lys192(conventional H-bond, carbon hydrogen bond),**<br>Val213 (alkyl)<br>**Gly216 (conventional H-bond)**	Ala190 (alkyl)<br>**Phe191 (unfavorable bump carbon hydrogen bond)**<br>**Lys192 (two alky)**<br>**Ser195 (conventional H-bond)**<br>Val213 (alky)<br>**Arg217 (conventional H-bond)**<br>**Ala226 (two alkyl)**
Patuletin (5281678)	Ala190 (conventional H-bond)<br>**Phe191 (amide-pi stacked)**<br>**Lys192 (pi-alkyl)**<br>**Ser195 (conventional H-bond)**<br>**Tyr215 (carbon-hydrogen bond, amide-pi stacked)**<br>**Gly216 (amide-pi stacked)**<br>**Arg217 (van der Waals)**<br>Ser218 (conventional H-bond)<br>**Ala226 (pi-alkyl)**<br>Val227 (carbon-hydrogen bond)	Ala190 (two conventional H-bond),<br>**Phe191 (amide-pi stacked),**<br>**Lys192 (pi-alkyl, pi-cation)**<br>**Tyr215 (amide-pi stacked)**<br>**Gly216 (carbon-hydrogen bond),**<br>Ser218 (conventional H-bond)	**Lys40 (unfavorable donor-donor)**<br>Ser189 (conventional H-bond)<br>Ala190 (conventional H-bond)<br>**Phe191 (pi-sigma, amide-pi stacked),**<br>**Lys192 (pi-alkyl),**<br>**Gly193 (conventional H-bond),**<br>**Ser195 (unfavorable acceptor-acceptor)**<br>Ser214 (conventional H-bond),<br>**Tyr215 (two amide-pi stacked),**<br>**Gly216 (two amide-pi stacked),**<br>**Arg217 (conventional H-bond)**<br>Ala220 (carbon-hydrogen bond),<br>Lys221 (two carbon-hydrogen bond)<br>Pro225 (two carbon-hydrogen bond)<br>**Ala226 (unfavorable bump, pi-alkyl)**
Salicylic Acid (338)	**Phe191 (amide-pi stacked)**<br>**Lys192 (van der Waals)**<br>**Ser195 (conventional H-bond)**<br>**Tyr215 (carbon-hydrogen bond, amide-pi stacked)**<br>**Gly216 (amide-pi stacked)**<br>**Ala226 (ai-alkyl)**	**Lys40 (salt bridge, two attractive charge)**<br>**His57 (pi-cation)**<br>**Lys192 (salt bridge, two attractive charge)**<br>**Ser195 (conventional H-bond)**	Ser189 (conventional H-bond)<br>Ala190 (conventional H-bond, pi-alkyl)<br>**Phe191 (amide-pi stacked),**<br>**Lys192 (van der Waals),**<br>**Tyr215 (amide-pi stacked)**<br>**Gly216 (carbon-hydrogen bond)**<br>**Arg217 (two conventional H-bond)**<br>**Ala226 (pi-alkyl)**

Umbelliferone (5281426)	**Phe191 (pi-sigma, amide-pi stacked)**<br>**Lys192 (van der Waals)**<br>**Tyr215 (amide-pi stacked)**<br>**Gly216 (van der Waals)**<br>**Arg217 (conventional H-bond)**<br>**Ala226 (pi-alkyl)**	Ala190 (conventional H-bond)<br>**Phe191 (two amide-pi stacked),**<br>**Lys192 (pi-alkyl),**<br>**Tyr215 (amide-pi stacked),**<br>**Gly216 (van der Waals)**	**Phe191 (pi-sigma, amide-pi stacked)**<br>**Lys192 (carbon-hydrogen bond)**<br>Ser214 (amide-pi stacked)<br>**Tyr215 (two amide-pi stacked)**<br>**Gly216 (van der Waals)**<br>**Arg217 (conventional H-bond)**<br>**Ala226 (two pi-alkyl)**
OHH (self-docking with crystallographic inhibitor)	**Lys40 (salt bridges, attractive charge, pi-cation)**<br>**His57 (pi-pi stacked)**<br>Cys58 (van der Waals)<br>Tyr96 (van der Waals)<br>Leu99 (van der Waals)<br>Ala190 (van der Waals)<br>**Phe191 (van der Waals)**<br>**Lys192 (amide-pi stacked),**<br>**Gly193 (conventional H-bond)**<br>**Asp194 (van der Waals)**<br>**Ser195 (conventional H-bond)**<br>Val213 (van der Waals)<br>Ser214 (van der Waals)<br>**Tyr215 (amide-pi stacked)**<br>**Gly216 (amide-pi stacked)**<br>**Arg217 (van der Waals)**<br>Ser218 (van der Waals)<br>**Ala226 (pi-alkyl)**	Thr36 (carbon-hydrogen bond, pi-sigma)<br>**Lys40 (pi-alkyl)**<br>**His57 (two pi-alkyl)**<br>Ala59 (conventional hydrogen bond)<br>**Phe191 (two amide-pi stacked),**<br>**Lys192 (conventional hydrogen bond, pi-alkyl)**<br>**Tyr215 (amide-pi stacked)**<br>**Gly216 (amide-pi stacked)**<br>**Arg217 (van der Waals)**	**Lys40 (conventional hydrogen bond, unfavorable bump, unfavorable positive-positive, salt bridge, two attractive charge)**<br>**His57 (carbon-hydrogen bond, two pi-pi stacked)**<br>Leu99 (pi-alkyl),<br>Ala190 (pi-alkyl),<br>Phe191 (amide-pi stacked),<br>**Lys192 (conventional hydrogen bond, alkyl, unfavorable bump, unfavorable positive-positive, two attractive charge)**<br>**Ser195 (unfavorable acceptor-acceptor)**<br>**Tyr215 (amide-pi stacked)**<br>**Gly216 (van der Waals)**<br>Ala220 (pi-alkyl)<br>**Ala226 (unfavorable bump, pi-alkyl)**
Methyllinderone (21953547)	**His57 (pi-lone pair interaction, pi-sigma interaction)**<br>**Lys192 (conventional H-bond, pi-alkyl interaction)**<br>Val213 (alkyl interaction)	Phe173 (pi-alkyl)<br>Ala190 (pi-alkyl)<br>**Val213 (alkyl)**<br>**Tyr215 (pi-alkyl)**<br>**Gly216 (conventional H-bond)**<br>**Ala226 (alkyl)**	**Phe191 (pi-alkyl)**<br>**Lys192 (alkyl)**<br>**Gly216 (conventional H-bond),**<br>**Arg217 (conventional H-bond, alkyl)**<br>**Ala226 (alkyl)**

Table S2. List of the feature mapping calculations performed on matricin, chlorogenic acid, and OHH (for comparison) in complex with chymase. The results were obtained starting from the best pose of the ligand as resulting from docking simulations for the two chamomile compounds, and on the crystallographic complex for OHH. Data for OHH were extracted from ref. [16].

**Compounds**	**Pharmacophore Features**
Matricin	HB_DONOR: 6<br>HB_ACCEPTOR: 13<br>HYDROPHOBIC: 2<br>TOTAL: 21
Chlorogenic acid	HB_DONOR: 13<br>HB_ACCEPTOR: 21<br>HYDROPHOBIC: 1<br>NEG_IONIZABLE: 1<br>RING_AROMATIC: 2<br>TOTAL: 38
OHH co-crystallized ligand	*HB_ACCEPTOR: 20*<br>*HYDROPHOBIC: 6*<br>*NEG_IONIZABLE: 2*<br>*RING_AROMATIC: 12*<br>*TOTAL: 40*

A)

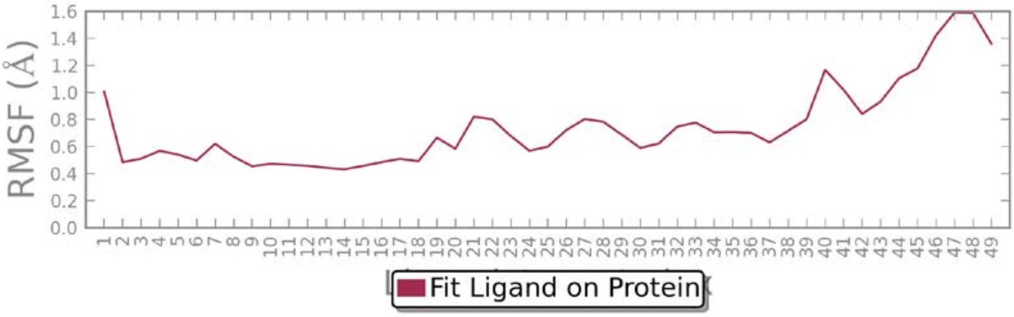

B)

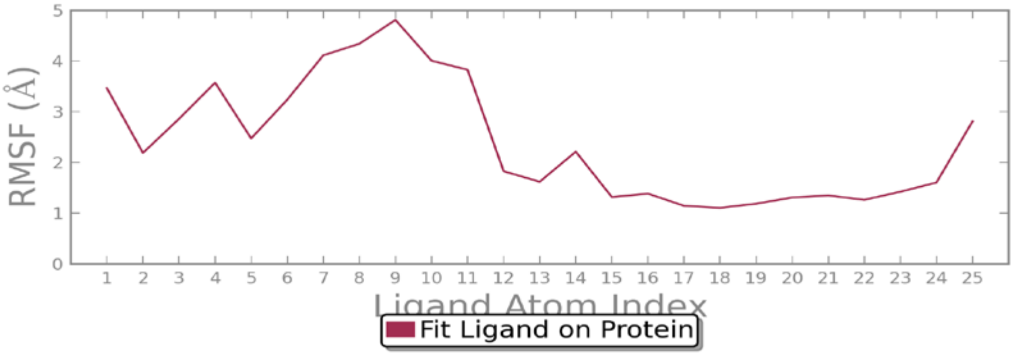

C)

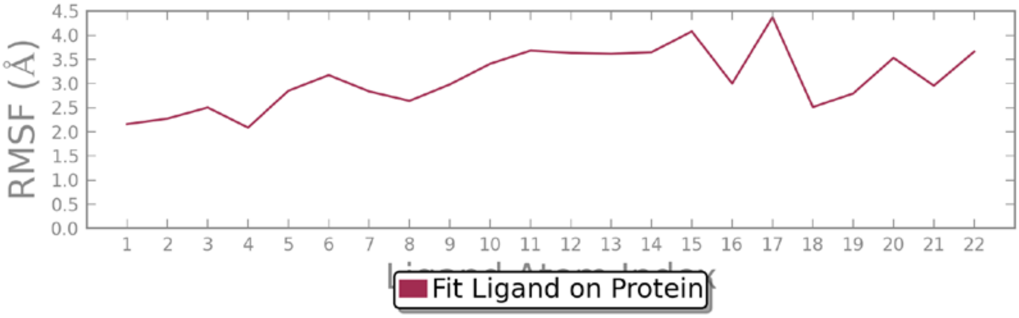

D)

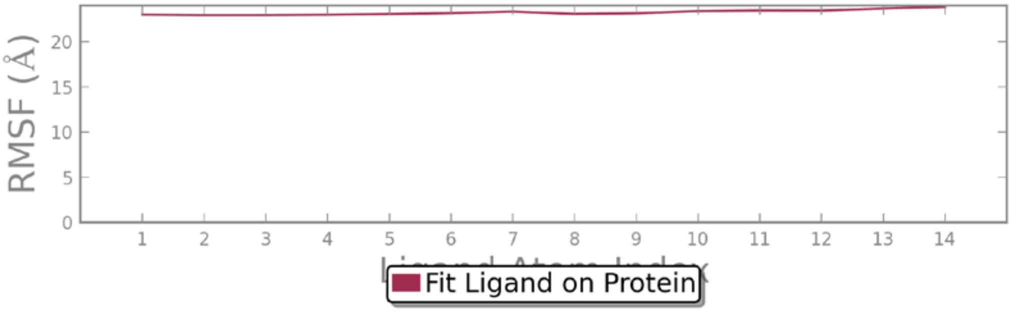

Figure S1. Ligand RMSF, measured on the ligand heavy atoms, of **(A)** OHH; **(B)** chlorogenic acid; **(C)** matricin; and **(D)** methyllinderone, compared to the starting structure.

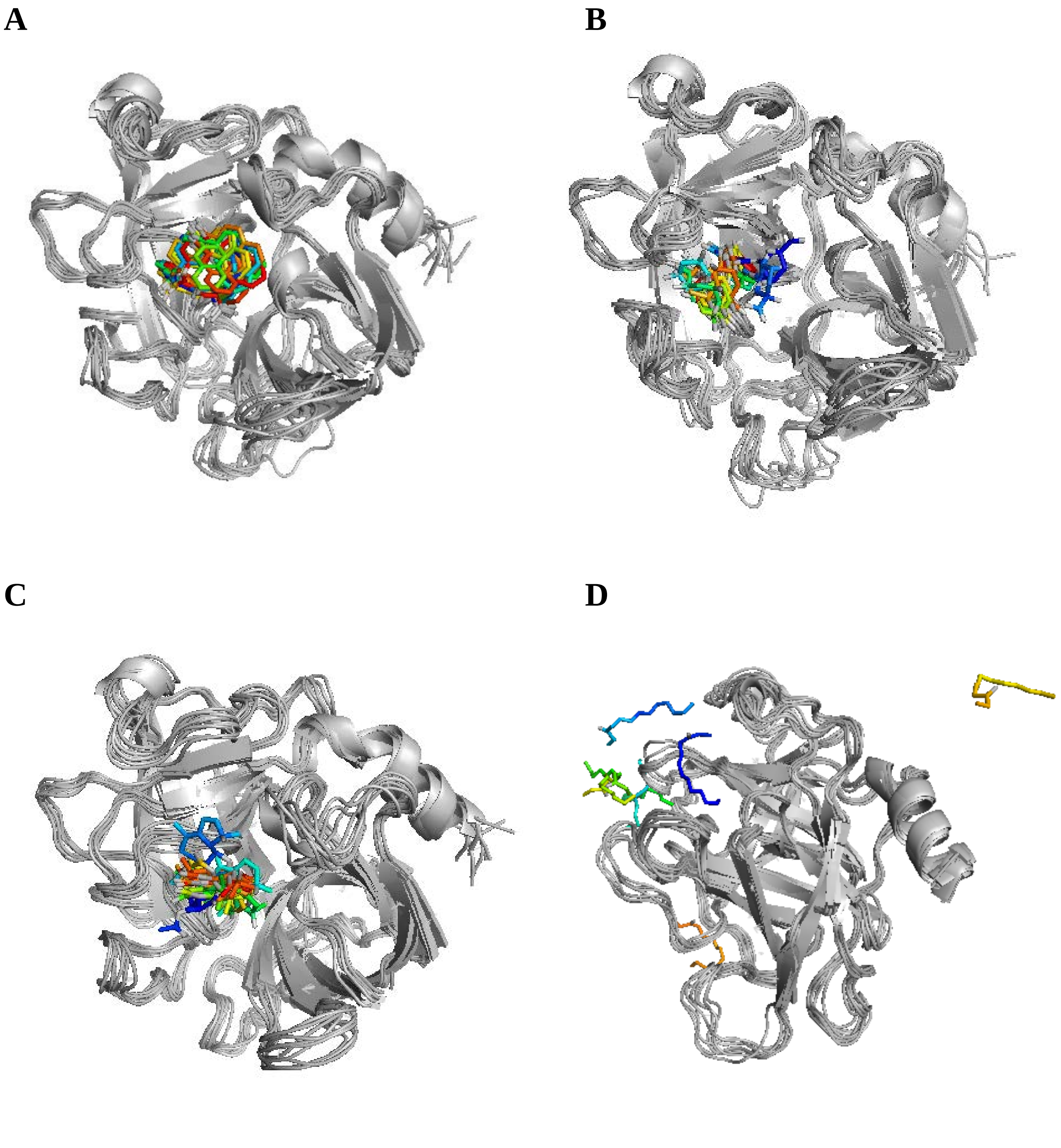

Figure S2: Snapshots of the positions of the ligands during the simulation for **(A)** OHH; **(B)** chlorogenic acid; **(C)** matricin; **(D)** methyllinderone. Snapshots were taken every 5 ns starting from *t* = 0. The colors of the ligands correspond to the different times.

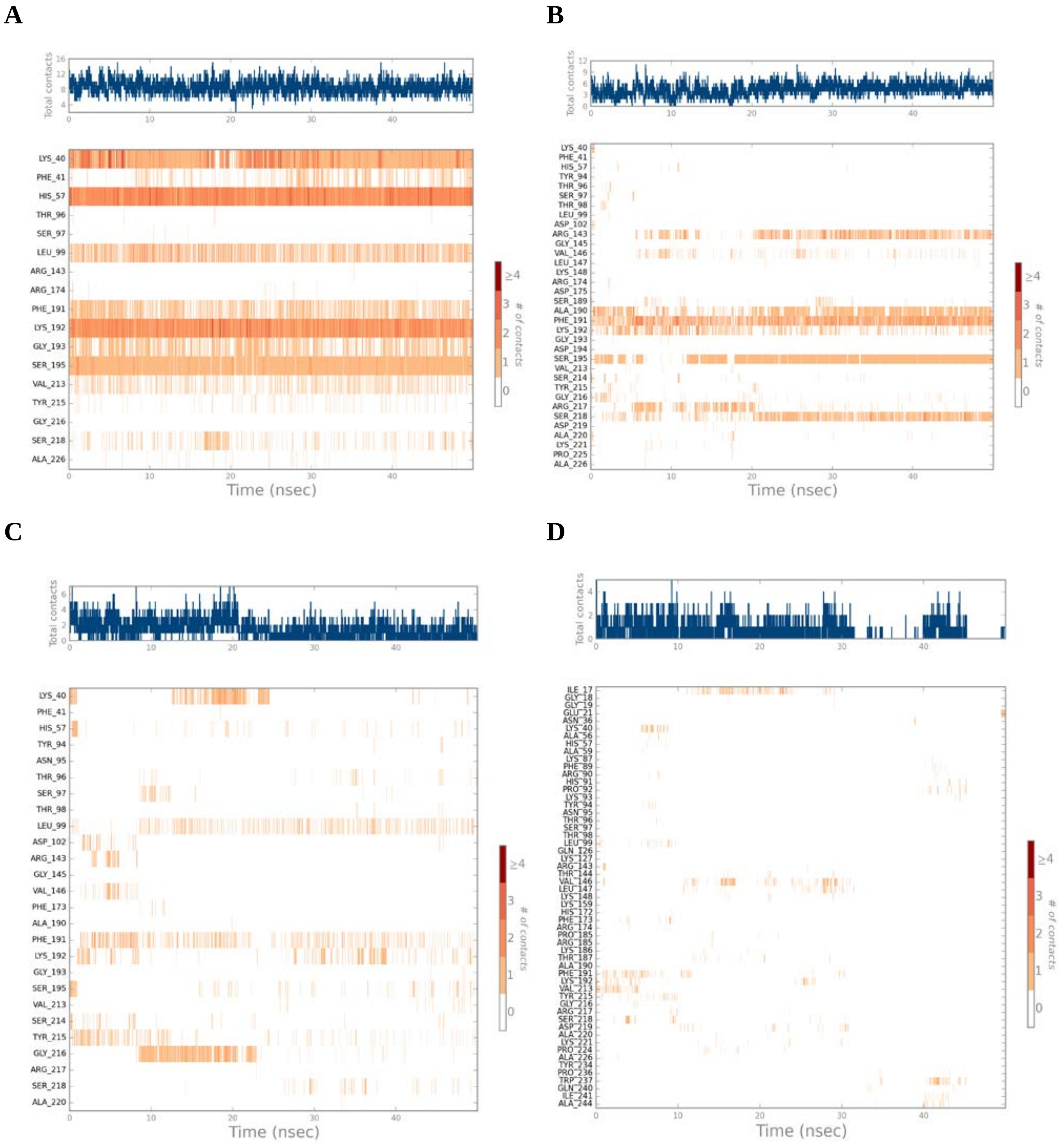

Figure S3. Timeline of protein–ligand contacts during the simulation for **(A)** OHH; **(B)** chlorogenic acid; **(C)** matricin; **(D)** methyllinderone. The top panel shows the total number of specific contacts the protein made with the ligand over the course of the trajectory. The bottom panel shows which residues interacted with the ligand in each trajectory frame. Some residues made more than one specific contact with the ligand, which is represented by a darker shade of orange, according to the scale to the right of the plot. The graphs were produced by Desmond analysis tools.
